# The Evolving Role of Multivitamin/Multimineral Supplement Use among Adults in the Age of Personalized Nutrition

**DOI:** 10.3390/nu10020248

**Published:** 2018-02-22

**Authors:** Jeffrey B. Blumberg, Regan L. Bailey, Howard D. Sesso, Cornelia M. Ulrich

**Affiliations:** 1 Friedman School of Nutrition Science and Policy, Tufts University, 711 Washington Street, Boston, MA 02111, USA; 2 Department of Nutrition Science, Purdue University, 700 West State Street, West Lafayette, IN 47907, USA; reganbailey@purdue.edu; 3 Brigham and Women’s Hospital and Harvard Medical School, 900 Commonwealth Avenue East, 3rd Floor, Boston, MA 02215, USA; hsesso@hsph.harvard.edu; 4 Huntsman Cancer Institute and Department of Population Health Sciences, University of Utah, 2000 Circle of Hope, Salt Lake City, UT 84112, USA; neli.ulrich@hci.utah.edu

**Keywords:** dietary supplement, nutrigenomics, deficiency diseases, micronutrients, nutrition, multivitamin

## Abstract

Micronutrient deficiencies occur in segments of the adult population in the United States. Multivitamin/multimineral supplements (MVMS) are widely used by this population, which reduces inadequacies in micronutrient intake, but the potential for exceeding tolerable upper intake levels in others should be considered. There are concerns associated with the excessive intake of certain nutrients, particularly folic acid, and potential untoward consequences. The advent of nutrigenomics and the enhanced ability to directly study the interactions between nutrition and genetic variants and expression will allow for the conduct of more targeted studies with specific endpoints and may ultimately lead to progress in the field of personalized nutrition. The role of MVMS in health maintenance and chronic disease prevention remains controversial. Conducting studies in this area has been hampered by, among other factors, inconsistent definitions of MVMS, ranging from as few as three vitamins to broad-spectrum products containing more than two dozen vitamins and minerals. Results from some observational studies and large-scale, randomized, controlled trials suggest that MVMS may reduce the risk of some forms of cancer and, potentially, cardiovascular disease. The ongoing COcoa Supplement and Multivitamin Outcomes Study (COSMOS) is expected to build on this research and provide additional insights into these areas.

## 1. Introduction

The World Health Organization has estimated that more than 2 billion people worldwide experience deficiencies in the intake of essential vitamins and minerals [1]. In the United States (US), a number of shortfall nutrients have been identified in the general population as described by the Dietary Guidelines Advisory Committee to the US Departments of Health and Human Services and Agriculture, which include vitamins A, C, D, and E and choline, calcium, magnesium, iron, and potassium [2]. Further, deficiencies in calcium, potassium, dietary fiber, and vitamin D are considered to be of public health concern based on their demonstrated role in health maintenance combined with their known low intake levels. Indeed, consistent with earlier reports, a recent analysis of National Health and Nutrition Examination Survey (NHANES) data indicates that a substantial number of individuals have intakes of these nutrients from dietary sources that fall below the Estimated Average Requirement (EAR) [3,4,5]. These dietary shortfalls occur despite the wide use of dietary supplements [6,7,8]. However, dietary supplements are often used by individuals who already have nutrient-rich diets. In particular among older women, multiple supplements can be used, which can increase the potential for oversupplementation and excessive nutrient intake [7,9]. Multivitamin/multimineral supplements (MVMS) are the most commonly utilized supplements among US adults, although their use overall has declined in recent years, from 37–40% in 1999–2006 to 31% in 2011–2012 [6].

The objective of this review is to summarize presentations on the patterns of MVMS use among adults from a scientific session of the American Society for Nutrition at the Experimental Biology 2017 Conference in Chicago, IL, USA. The session reviewed the role of MVMS and described evidence from observational studies and randomized, controlled trials that evaluated the effects of MVMS on chronic disease outcomes. The evolving field of nutrigenomics and its influence on the application of personalized nutrition was also discussed.

## 2. Role of MVMS in the US Diet

Approximately half of all US adults take some form of dietary supplement, with vitamin and mineral supplements accounting for a substantial portion of total use [6,9]. Despite a recent apparent decrease in MVMS use overall, the most recent NHANES data (2011–2014) suggest that 34–49% of older adults regularly take an MVMS [10]. As of April 2017, the Dietary Supplement Label Database of the US National Institutes of Health Office of Dietary Supplements and National Library of Medicine listed 1404 different vitamin/mineral products containing the word “multi” [11]. 

Estimating the use and evaluating the benefits and risks of MVMS in observational studies and controlled trials is complicated by a lack of a consistent scientific or regulatory definition of these over-the-counter products [12]. Definitions of MVMS vary according to investigators, professional organizations, and manufacturers. For example, different NHANES analyses have included MVMS products containing ≥3 vitamins, ≥3 vitamins plus ≥1 mineral, and ≥9 or ≥10 total micronutrients [4,6,9,13,14]. The Older Americans Act Amendment of 2006 proposed that an MVMS should contain at least two-thirds of vitamins and essential minerals and provide 100% of the Daily Value (DV) for the intended life stage [15], but official definitions of MVMS continue to evolve [16]. Some MVMS are formulated to contain approximately 100% of the DV for as many as 30 vitamins and essential minerals [17,18,19], while other products have been designed to address a particular health problem, such as those used for maintaining eye health in older adults [20]. Notably, the health claims of dietary supplements are regulated as foods, not as pharmaceuticals, by the US Food and Drug Administration (FDA) [21]. These definitions also do not generally account for micronutrient inadequacies or needs based on factors such as age, health status, or dietary pattern, which are each pertinent considerations when formulating an MVMS in the context of personalized nutrition.

In reports analyzing NHANES data, the most common reasons cited by consumers for using an MVMS were to maintain or improve overall health, prevent health problems, and promote bone or heart health [10,22]. In these two studies, only 22% of individuals reported using these products specifically to supplement their diets. Importantly, individuals with healthier lifestyles were shown to be more likely to use MVMS [22], and analyses of NHANES data from 2003 to 2006 found that supplement users had comparatively higher intakes of most vitamins and minerals from their dietary choices alone than did those who reported not using supplements [7,8]. These factors can lead to intakes exceeding the tolerable upper intake level (UL) in some users, while those who are not reaching adequate intake levels from their diets are also less likely to be using dietary supplements to reach those levels.

Given the potential of micronutrient inadequacies or deficiencies in certain segments of the adult US population [3,4,5], approaches to addressing these issues, beyond general nutrition education, should be considered. Deficiencies in micronutrient intake are related to the socioeconomic status, with a significant association being observed between income and micronutrient intake, as well as dietary supplement use [23]. Adults in higher income brackets had a lower prevalence of inadequate intakes compared with those in lower income brackets. Therefore, strategies to enhance nutrient intakes across certain segments of the US population may be necessary, while also considering the risk of exceeding ULs in those already achieving an adequate intake from their diets and/or taking multiple dietary supplements. For individuals at risk of inadequate consumption, dietary supplements may be used to fill gaps in nutrient intake without increasing caloric intake.

**Figure 1:**
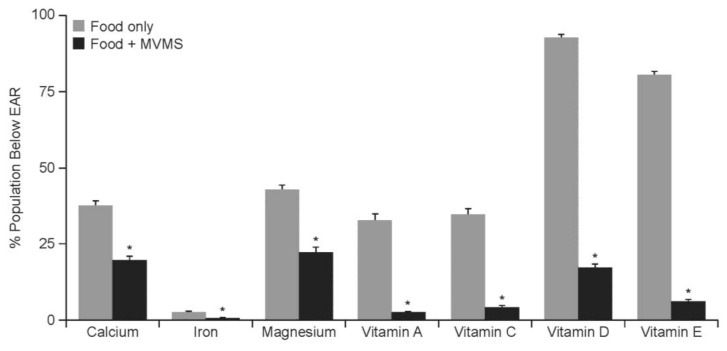
Proportion of subjects ≥19 years of age achieving an intake of shortfall nutrients below the EAR from food or from food + MVMS. Reprinted from Blumberg, J.B., et al. [3]. Abbreviations: EAR: Estimated Average Requirement; MVMS: multivitamin/multimineral supplement. * *p* < 0.01 versus food only.

Evidence that dietary supplement use increases micronutrient intake includes analyses of NHANES data collected between 2009 and 2012. These analyses revealed that MVMS use by individuals ≥19 years of age significantly reduced the proportion of subjects with intakes below the EAR for 15 of the 17 micronutrients evaluated, including for 7 of the 10 nutrients that are deemed “underconsumed” in the 2015–2020 Dietary Guidelines for Americans (Figure 1) [3].

**Figure 2:**
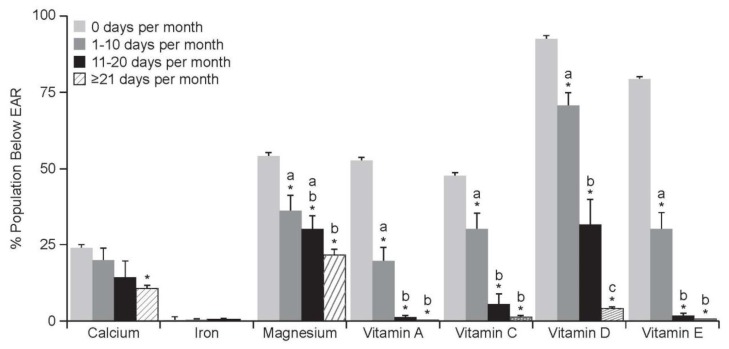
Proportion of subjects ≥19 years of age achieving an intake of shortfall nutrients below the EAR from food + MVMS by frequency of intake. Reprinted from Blumberg, J.B., et al. [3]. Abbreviations: EAR: Estimated Average Requirement; MVMS: multivitamin/multimineral supplement. * *p* < 0.01 versus 0 days per month; ^a,b,c^ Values by frequency of MVMS use with different superscripts are significantly different (*p* < 0.01).

The greatest decrease in individuals with intakes below the EAR was observed in those whose frequency of MVMS use was ≥21 days/month (Figure 2).

It is noteworthy that MVMS use in this specific analysis significantly increased the prevalence of intake above the UL in ≤4% of the population for seven nutrients. However, further research is warranted to determine whether any untoward outcomes are associated with even this low level of overconsumption. In addition to dietary supplements, the nutritional status can also be improved by consuming micronutrient-fortified foods; NHANES data have shown that the consumption of highly fortified breakfast cereals can fill many gaps in micronutrient intake [24].

In summary, accurately assessing the role of MVMS is complicated by the lack of a consistent definition for this product category, but data clearly suggest that using an MVMS can improve the nutritional status. However, the future of supplementation may involve developing new, targeted formulations such as those designed to provide more personalized nutrition (e.g., those that focus on the interactions between risk factors for chronic disease, life stage, nutrient intake, and genetics). Determining the presupplementation levels of dietary nutrient intake should also be considered to ensure that individuals are not exceeding the UL.

## 3. Micronutrient Intake/Status and Gene Expression

The sequencing of the human genome has allowed for identifying interactions between nutrient intake and the activity of specific genes related to health, which has given rise to the field of nutrigenomics [25,26]. Nutrigenetics and nutrigenomics are the terms that are used to describe the science that evaluates the impact that genetic variants have on the dietary response and determines the effect that nutrients and bioactive food compounds may have on gene expression, respectively [27]. Multiple single-nucleotide polymorphisms (SNPs) appear to affect the synthesis and function of key proteins, with implications for the modification of nutrient requirements and metabolism [26]. This interaction between nutrition, gene expression, and health has been well established for lactose intolerance and phenylketonuria [26], but emerging research suggests an array of other targets, including the risk for developing chronic diseases. Although controversy exists regarding the current applicability of genetic-based personalized nutrition [28], it is important to note that clinical trials suggest that behavioral coaching informed by personal data may improve the adherence to dietary advice and clinical biomarkers [29,30], and businesses are now offering relevant services to practitioners [31]. Some studies have investigated genome-wide predictors of nutrient levels in plasma or serum. Although statistically significant genetic polymorphisms have been identified as predictors, they have explained little variation in nutrient levels thus far [32]. For example, vitamin D heritability is only 7.5%, and of that, only 38% can be explained by known genetic variants; this represents only 3% of the total variability in circulating 25-OH-D levels [33].

Folic acid has been well studied with regard to nutrigenomic interactions involving the one-carbon metabolism pathway central to DNA methylation and nucleotide synthesis [34,35,36,37]. Furthermore, the link between folate deficiencies or inadequacies and the occurrence of neural tube defects (NTDs) and, conversely, folic acid supplementation during pregnancy and reduction in the occurrence and reoccurrence of NTDs has been firmly established. As a result, folic acid supplementation has become routine during pregnancy [38,39,40]. However, folate may also play a role in the pathogenesis of a number of other health problems, including cardiovascular disease (CVD), cancer, and neurodegenerative conditions such as dementia and Alzheimer’s disease [41,42,43,44,45,46,47,48,49,50].

Methylenetetrahydrofolate reductase (MTHFR) catalyzes the conversion of 5,10-methylenetetrahydrofolate to 5-methylenetetrahydrofolate, an irreversible step in folate metabolism [51,52,53,54]. Molecular epidemiology studies have found that the C677T polymorphism in the MTHFR gene may modulate the risk of colorectal cancer [51,52,53,54]. The direction and magnitude of this risk modification is influenced by folate status [53,54], as well as by alcohol consumption [52] and the supply of methyl group donors [51]. A study conducted in women found that the C677T polymorphism was further associated with a 62% increased risk of breast cancer; however, this risk was ameliorated by the intake of nutrients involved in one-carbon metabolism, including folate and related B vitamins, as well as the amino acid methionine [55].

Despite the promising evidence related to folate status and positive health outcomes, there are also data suggesting that folic acid supplementation in some individuals can cause adverse health outcomes. Results from the Aspirin/Folate Polyp Prevention Study revealed a trend toward an increased risk of advanced colorectal lesions and adenoma multiplicity among subjects randomized to receive folic acid supplementation [56]. Another study also found that folic acid supplementation was associated with a significant increase in the risk of prostate cancer (hazard ratio (HR): 2.63; 95% confidence interval (CI): 1.23–5.65) [57]. Further, in the Women’s Health Initiative (WHI) study, women with high folate status during the period prior to routine folic acid fortification had lower levels of DNA methylation than in the postfortification period. These results suggest that the relationship between folate status and DNA methylation is not linear and that fortification in otherwise well-nourished individuals may attenuate the positive effects of folate and cause adverse health outcomes [58]. Other evidence also supports the possibility that folic acid supplementation in the presence of fortification may cause excess dietary intake and increase the risk of developing some forms of cancer [56,57,59]. Overall, a dual role of folate in carcinogenesis has been proposed and substantiated by mathematical modeling [59,60]. Variants in genes other than MTHFR associated with folate and one-carbon metabolism have been linked to both increases and decreases in the risk of colorectal cancer, depending on folate status [37]. Therefore, clinicians must evaluate potential risks as well as benefits when considering folic acid supplementation in individuals who may be at risk for certain types of cancer.

In addition to the effects of folate status on DNA methylation and the development of some types of cancer, there are also data implicating folate status in a number of other biological processes and disease outcomes. A study investigating predictors of long interspersed nucleotide element (LINE) methylation noted that folate status seemed to modify the associations, in particular, those observed between sex hormones and DNA methylation [61]. Unmetabolized folic acid has been linked to reduced natural killer cell cytotoxicity in both humans and mice [62,63]. Other studies have suggested a role for folate–gene interactions in inflammation, diabetes, and the health of newborns [64,65,66].

Interactions between MTHFR polymorphisms and other genes with folate and vitamin B_12_ status may also affect the development of Alzheimer’s disease [67]. In one study, high dietary folate levels were not found to be beneficial to memory in community-dwelling, elderly subjects with a del/del 19-bp polymorphism in the DHFR gene, which codes dihydrofolate reductase [68]. Indeed, higher dietary folate levels may cause adverse effects on cognitive functioning. Variants in the MAT1A gene, which is responsible for coding methionine adenosyl transferase, have also been linked to hypertension and stroke; however, depending on the individual’s genotype, improving vitamin B_6_ status might decrease this risk [69].

Other nutrient–gene interactions relevant to health outcomes have also been identified. For example, vitamin D inadequacy has been linked to increased cancer risk and/or tumor development through regulation of gene expression relating to cell cycle progression, apoptosis, cellular adhesion, oxidative metabolism, immune function, and steroid metabolism [70,71]. Vitamin D has also been implicated in the transcriptional activation of tryptophan hydroxylase-2, which catalyzes serotonin synthesis and thus may have relevance to some neurological conditions, such as attention deficit hyperactivity disorder, bipolar disorder, schizophrenia, depression, impulsive behavior, and autism [72,73]. However, clinical benefits have not been demonstrated in controlled trials of the impact of vitamin D supplementation (alone or with calcium) on neurological conditions [74,75]. Evidence is also available for other gene–nutrient interactions, which, despite being limited, warrant mentioning. For example, polymorphisms in the vitamin C co-transporter gene have been shown to be associated with an increased risk of primary open-angle glaucoma [76]. Additionally, the ability of vitamin E supplementation to prevent respiratory tract infections in the elderly has been suggested to be dependent on interleukin-10 SNPs for immunoregulatory genes [77]. Interactions have also been observed between vitamin E and polymorphisms controlling the production of other cytokines, including tumor necrosis factor-α, which may impact the immunomodulatory effects of vitamin E supplementation [78].

As knowledge of nutrient–gene interactions and their relationship to intermediary disease biomarkers evolves, nutrigenomics has the potential to substantially inform personalized nutrition and help individualize recommendations for the use of dietary supplements. Using a targeted approach in this field will allow for the conduct of clinical trials focused on those populations most likely to experience significant benefits on specific endpoints based on their genotype. Such studies will also help identify likely “nonresponders,” as well as those susceptible to untoward outcomes from supplementation with specific nutrients.

In addition to the impact of genetic factors on the nutritional status, in numerous other individuals, common factors also impact the absorption, metabolism, distribution, utilization, storage, and excretion of nutrients obtained from MVMS. Nonmodifiable factors that may alter the nutrient status include age, sex, and environmental toxicants [79]. Modifiable factors that have previously been demonstrated to alter nutrient absorption include smoking [80], medication use (i.e., drug–nutrient interactions) [81,82,83], nutrient intake (i.e., nutrient–nutrient interactions), inflammation [84], life-stage (e.g., pregnancy, lactation), and presence of disease. Furthermore, the increasing body weight is a significant public health challenge [85], and a growing body of scientific literature suggests that a suboptimal weight status is associated with a poor micronutrient status [80,86]. For example, obesity is associated with poor vitamin D status [87], and obese women of childbearing potential are at higher risk for poor folate status [88]. Additionally, obese adults are less likely to use dietary supplements than those within their recommended body mass index range [9,22].

## 4. Emerging Evidence on MVMS in Chronic Disease Outcomes

Observational studies and randomized clinical trials of MVMS offer optimal settings in which to examine important nutrient–gene interactions and other promising areas of nutrigenomics to enable the personalization of recommendations for MVMS use across a range of populations and chronic disease outcomes. However, no observational studies or randomized clinical trials of MVMS have considered either confounding or effect modification by genetic variation, which may be an important consideration in the results from observational studies on MVMS described below. While a well-conducted clinical trial offers the key advantage of eliminating unmeasured confounding, there may be important modifying effects by genetic variation that build upon one-carbon metabolism and other important mechanistic pathways related to nutrigenomics.

### 4.1. Observational Studies

The outcomes of observational studies have revealed inconsistent results regarding the benefits of MVMS in the prevention of chronic diseases. For example, the Cancer Prevention Study II, conducted in a population of more than 1 million Americans, reported a reduction in the risk of cardiovascular (CV) mortality in users of MVMS and vitamin A, C, or E supplements [89]. However, the same study reported an increase in the risk of cancer mortality among male supplement users who smoked. The Stockholm Heart Epidemiology Program found a reduction in nonfatal myocardial infarction in both occasional and regular users of dietary supplements (including MVMS and single-nutrient supplements) compared with nonusers [90], which was supported by similar results among women in the Swedish Mammography Cohort [91]. The WHI reported no overall association between the use of MVMS and the risk of CVD in postmenopausal women [92]. However, in a prospective analysis of NHANES data, a protective association was observed between MVMS use over an 18 year follow-up period and CVD-related mortality in a population of women [93].

Observational studies that have evaluated MVMS in cancer risk have primarily reported null results. As observed for CVD, the WHI found no association between MVMS use and the risk of cancer at several common sites [92]. The Multiethnic Cohort Study also found no significant relationship between MVMS use and cancer risk, either overall or at specific sites [94]. However, a prospective analysis of the Cancer Prevention Study II cohort found an 11% reduction (relative risk (RR): 0.89; 95% CI: 0.80–0.99) in colorectal cancer among individuals taking an MVMS that was presumed to contain folic acid [95]. 

All observational studies of MVMS have several inherent limitations that preclude making definitive conclusions about the risk of various chronic disease endpoints, including inconsistent definitions of MVMS, imprecision regarding the frequency and duration of MVMS use, and residual confounding. Further, many observational studies of MVMS use lack data on baseline micronutrient status, changes in dietary patterns during the follow-up period, as well as the potential reasons for these changes (i.e., the development of new comorbidities). It is also possible, as suggested by the long-term follow-up of the Physicians’ Health Study (PHS) I that evaluated CVD outcomes [96], that MVMS use may need to be of sufficient duration to observe a significant health benefit.

### 4.2. Randomized, Controlled Trials

In addition to these observational data, a number of randomized, controlled clinical trials that evaluated MVMS have been conducted; some key studies are summarized in Table 1 [17,18,19,20,97,98,99,100,101,102,103,104,105].

The Linxian trial was conducted in subjects with a diagnosis of esophageal dysplasia and found no overall reduction in the risk of cancer for those randomized to receive MVMS or placebo. However, a nonsignificant 8% reduction in esophageal/cardia cancer mortality (RR: 0.92; 95% CI: 0.67–1.28) and a nonsignificant 38% reduction in cerebrovascular mortality (RR: 0.62; 95% CI: 0.37–1.06) were observed [98]. In the Supplémentation en Vitamines et Minéraux Antioxydants (SU.VI.MAX) study, which randomized men and women to receive a combination of antioxidants, including ascorbic acid, vitamin E, β-carotene, selenium, and zinc, or placebo, there was a significant reduction in the risk of cancer in men (RR: 0.69; 95% CI: 0.53–0.91) [101]. After a median of 7.54 years of follow-up, there were no differences in the incidence of all-cause cancer or ischemic CVD; however, interestingly, a post hoc analysis of the SU.VI.MAX study revealed that 5 years after the end of the trial, MVMS use among men was associated with a significantly greater probability of healthy aging (RR: 1.16; 95% CI: 1.04–1.29) [106]. After a median follow-up of 11.2 years, the randomized, double-blind, placebo-controlled PHS II observed a significant 8% reduction in total cancer in subjects who received MVMS compared with those who received the placebo (HR: 0.92; 95% CI: 0.86–0.998) [100]. Additionally, MVMS use was associated with a reduction in cancer risk of 18% in those ≥70 years of age (HR: 0.82; 95% CI: 0.72–0.93) and a reduction of 27% among those with histories of cancer (HR: 0.73; 95% CI: 0.56–0.96; *p* = 0.02) [100]. 

In contrast to the results related to cancer risk, the PHS II study found no impact of MVMS use on the risk of major CV events (HR: 1.01; 95% CI: 0.91–1.10) [18]. There was, however, a significant reduction in myocardial infarction-related death (HR: 0.56; 95% CI: 0.33–0.95), but this finding may be the result of chance, as a total of only 70 events were observed [18]. In secondary analyses of the PHS II trial results on MVMS use, men aged ≥70 years who consumed a healthy diet (based on Alternative Healthy Eating Index and Alternate Mediterranean Diet Score) appeared to benefit from taking an MVMS on the basis of the observed reduction in the incidence of myocardial infarction [107]. A smaller short-term clinical trial also demonstrated a positive effect of MVMS on changes in CV risk factors in obese women [17,19], but larger studies are needed to further investigate additional outcomes related to CV health. The results of these studies highlight the potential for targeted nutrition strategies that may or may not include an MVMS that is tailored to an individual’s life stage, chronic disease history, or nutritional status.

There do not appear to be any major safety concerns associated with the long-term use of broad-spectrum MVMS because their use does not significantly increase the risk of exceeding the UL [3,108]. A systematic review of 15 MVMS studies reported that only mild gastrointestinal adverse events were observed [108]. In the randomized, controlled PHS II, MVMS use did not produce significant effects on gastrointestinal side effects, fatigue, drowsiness, skin discoloration, or migraine [18]. However, MVMS use was associated with a modest increase in skin rashes, as well as some inconsistent effects on minor bleeding that were considered to be more likely a function of chance than effect [18].

Meta-analyses have provided additional ambiguity regarding the role of MVMS for health promotion. A meta-analysis of randomized, controlled trials by MacPherson et al. [109] indicated that using an MVMS had no effect on overall mortality (RR: 0.98; 95% CI: 0.94–1.02), but in the primary prevention studies there was a modest trend toward a reduction in the risk of all-cause mortality (RR: 0.94; 95% CI: 0.89–1.00). A key weakness of this analysis, however, was the wide variability in the definitions of MVMS that were combined across a wide array of studies included in the meta-analysis; any vitamin/mineral product with three or more ingredients (excluding B vitamin-only combinations) was analyzed [109]. As a result, the US Preventive Services Task Force indicated that there is insufficient evidence to recommend using an MVMS in the prevention of cancer, CVD, or mortality [110]. Table 1 illustrates this heterogeneity in the design of MVMS studies, with different definitions of MVMS, subject inclusion criteria, and length of follow-up. Therefore, interpreting the total body of evidence from randomized, controlled trials conducted with MVMS remains difficult.

Because of the limited number of long-term randomized, controlled trials of MVMS but given the potential benefits suggested by the PHS II [99,100] and several large scale, prospective observational studies, additional research is warranted. The forthcoming randomized, controlled COcoa Supplement and Multivitamin Outcomes Study (COSMOS) has been designed to evaluate an MVMS and a cocoa extract (containing 600 mg cocoa flavanols) on the incidence of major CV outcomes and invasive cancer [111]. COSMOS plans to enroll 12,000 women ≥65 years of age and 6000 men ≥60 years of age who are free of CVD or were not recently diagnosed with cancer. Subjects will be randomized in a 2 × 2 factorial design to receive the cocoa extract (600 mg) or a placebo and an MVMS (including 30 essential vitamins, minerals, and bioactives) or a placebo. The primary clinical endpoints of COSMOS are major CV events (myocardial infarction, stroke, CVD-related death, and coronary revascularization) and invasive cancer [111].

## 5. Conclusions

The use of dietary MVMS is common among adults in the US, and using an MVMS has been shown to reduce the prevalence of inadequate micronutrient intake (i.e., intakes below the EAR) and status. Exceeding the UL, however, should be considered in vulnerable subgroups such as children and older adults and those already taking multiple dietary supplements. High dietary intakes from supplements with certain nutrients, in particular, folic acid, may have negative health outcomes such as increasing the progression of precancerous lesions and tumors. Nutrigenomic approaches should provide new insights into the pursuit of providing personalized nutrition, as ongoing research continues to elucidate the role of nutrition in gene expression and disease. Evidence from some large prospective, cohort studies and randomized, controlled trials suggests that MVMS may contribute to a reduction in the risk of some chronic diseases such as CVD and cancer, but additional long-term clinical trials are necessary. Agreement on the standardization of MVMS products by the FDA would provide a helpful contribution to future research studies testing these products. Forthcoming studies assessing the health benefits and risks of MVMS and other dietary supplements should involve specific objectives and methods relevant to individualizing outcomes and informing the practice of personalized nutrition. 

## Figures and Tables

**Table 1:** Randomized, controlled trials of MVMS.

Reference	Study Name	Number of Subjects	Population	MVMS Formulation	Endpoints	Study Duration	Results
Blot, et al. [97]	NA	29,584	Adults from rural Linxian, China, 40–69 years of age	β-Carotene, selenium, α-tocopherol	Mortality, cancer incidence	5.25 years	9% reduction in overall mortality, 13% reduction in cancer mortality, 7% reduction in overall cancer incidence versus nonuse
Li, et al. [98]	NA	3318	Adults from rural Linxian, China, 40–69 years of age with a diagnosis of esophageal dysplasia and low dietary intake of several nutrients	Broad-spectrum MVMS*	Mortality, cancer incidence	6 years	No significant effect on mortality or cancer incidence, 38% nonsignificant reduction in cerebrovascular death (*p* = 0.08) versus placebo
AREDS [103,104]	AREDS	4757	US adults 55–80 years of age with evidence of grade 1–4 AMD	Antioxidants (vitamin C 500 mg, vitamin E 400 IU, β- carotene 15 mg), zinc oxide 80 mg (plus cupric oxide 2 mg)	Incidence of advanced AMD, incidence of cataract	6.3 years	Significant reduction in risk of progression to advanced AMD with antioxidants + zinc (OR: 0.72; 95% CI: 0.52–0.98; *p* = 0.007) versus placebo, no difference in incidence of lens opacities versus nonuse
AREDS2 [20,105]	AREDS 2	4203 subjects with risk of progression in 6916 eyes	US adults 50–85 years of age at high risk of progression to advanced AMD	Lutein 10 mg + zeaxanthin 2 mg, DHA 350 mg + EPA 650 mg. All subjects received the AREDS formulation (vitamin C, vitamin E, β-carotene, zinc, and copper; see AREDS above for doses)	Incidence of advanced AMD, progression to cataract surgery (comparison for lutein + zeaxanthin versus placebo only)	5 years	No significant difference versus placebo in progression to advanced AMD or cataract surgery
Christen, et al. [99]; Sesso, et al. [18]; Gaziano, et al. [100]	PHS II	14,641	US male physicians ≥50 years of age	Broad-spectrum MVMS *	Cancer incidence, MACE, incidence of cataract and AMD	11.2 years	8% reduction in total cancer incidence (*p* = 0.04) versus placebo, no effect on MACE versus placebo, 9% reduction in risk of cataract (*p* = 0.04) versus placebo, nonsignificant 19% increase in AMD versus placebo
Hercberg, et al. [101]	SU.VI.MAX	13,017	French women 35–60 years of age and men 45–60 years of age	Ascorbic acid 120 mg, vitamin E 30 mg, β-carotene 6 mg, selenium 100 µg, zinc 20 mg	Major fatal and nonfatal ischemic CV events, cancer incidence	7.54 years	No significant effect on CV outcomes or overall cancer incidence, significant reduction in cancer incidence in men (*p =* 0.008) versus placebo
Lamas, et al. [102]	TACT	1708	US adults ≥50 years of age and sustained myocardial infarction ≥6 weeks prior to enrollment	28-Component mixture reflecting that used by chelation practitioners	Composite of time to death from any cause, re-infarction, stroke, coronary revascularization, or hospitalization for angina	55 months	Nonsignificant 11% reduction in risk of death or CV events versus placebo
Wang, et al. [19]; Li, et al. [17]	NA	96	Obese Chinese women 18–55 years of age	Broad-spectrum MVMS *	Anthropometry, blood pressure, resting energy expenditure, biochemistry	26 weeks	Significant reductions in body weight, BMI, fat mass, systolic and diastolic blood pressure, total cholesterol, LDL-cholesterol, and significant increase in resting energy expenditure and HDL-cholesterol versus placebo

Abbreviations: AMD: age-related macular degeneration; AREDS: Age-Related Eye Disease Study; BMI: body mass index; CI: confidence interval; CV: cardiovascular; DHA: docosahexaenoic acid; EPA: eicosapentaenoic acid; HDL: high-density lipoprotein; LDL: low-density lipoprotein; MACE: major cardiovascular event; MVMS: multivitamin/multimineral supplement; NA: not applicable; OR: odds ratio; PHS: Physicians’ Health Study; SU.VI.MAX: Supplémentation en Vitamines et Minéraux Antioxydants; TACT: Trial to Assess Chelation Therapy; US: United States. * MVMS containing 26–30 different vitamins, minerals, and bioactives at amounts that primarily meet or slightly exceed daily values based on the study population [18,19,98].

## References

[1] Allen L., de Benoist B., Dary O., Hurrell R. (2006). Guidelines on Food Fortification with Micronutrients.

[2] U.S. Department of Agriculture (2015). Dietary Guidelines for Americans 2015–2020.

[3] Blumberg J.B., Frei B.B., Fulgoni V.L., Weaver C.M., Zeisel S.H. (2017). Impact of frequency of multi-vitamin/multi-mineral supplement intake on nutritional adequacy and nutrient deficiencies in U.S. adults. Nutrients.

[4] Wallace T.C., McBurney M., Fulgoni V.L.I. (2014). Multivitamin/mineral supplement contribution to micronutrient intakes in the United States, 2007–2010. J. Am. Coll. Nutr..

[5] Fulgoni V.L.I., Keast D.R., Bailey R.L., Dwyer J. (2011). Foods, fortificants, and supplements: Where do Americans get their nutrients?. J. Nutr..

[6] Kantor E.D., Rehm C.D., Du M., White E., Giovannucci E.L. (2016). Trends in dietary supplement use among US adults from 1999-2012. JAMA.

[7] Bailey R.L., Fulgoni V.L., Keast D.R., Dwyer J.T. (2011). Dietary supplement use is associated with higher intakes of minerals from food sources. Am. J. Clin. Nutr..

[8] Bailey R.L., Fulgoni V.L.I., Keast D.R., Dwyer J.T. (2012). Examination of vitamin intakes among US adults by dietary supplement use. J. Acad. Nutr. Diet..

[9] Bailey R.L., Gahche J.J., Lentino C.V., Dwyer J.T., Engel J.S., Thomas P.R., Betz J.M., Sempos C.T., Picciano M.F. (2011). Dietary supplement use in the United States, 2003–2006. J. Nutr..

[10] Gahche J.J., Bailey R.L., Potischman N., Dwyer J.T. (2017). Dietary supplement use was very high among older adults in the United States in 2011–2014. J. Nutr..

[11] Dietary Supplement Label Database. https://dsld.nlm.nih.gov/dsld/rptQSearch.jsp?item=multi&db=adsld.

[12] Yetley E.A. (2007). Multivitamin and multimineral dietary supplements: Definitions, characterization, bioavailability, and drug interactions. Am. J. Clin. Nutr..

[13] Radimer K., Bindewald B., Hughes J., Ervin B., Swanson C., Picciano M.F. (2004). Dietary supplement use by US adults: Data from the National Health and Nutrition Examination Survey, 1999–2000. Am. J. Epidemiol..

[14] Bailey R.L., Gahche J.J., Thomas P.R., Dwyer J.T. (2013). Why US children use dietary supplements. Pediatr. Res..

[15] 109th US Congress. Older Americans Act Amendments of 2006. http://www.gpo.gov/fdsys/pkg/PLAW-109publ365/html/PLAW-109publ365.htm.

[16] Global Health and Nutrition Network Reps. Love, Cardenas Explain Support for SNAP Multivitamin legislation. https://www.naturalproductsinsider.com/blogs/insider-law/2017/10/rep-love-explains-support-for-snap-multivitamin-l.aspx.

[17] Li Y., Wang C., Zhu K., Feng R.N., Sun C.H. (2010). Effects of multivitamin and mineral supplementation on adiposity, energy expenditure and lipid profiles in obese Chinese women. Int. J. Obes..

[18] Sesso H.D., Christen W.G., Bubes V., Smith J.P., MacFadyen J., Schvartz M., Manson J.E., Glynn R.J., Buring J.E., Gaziano J.M. (2012). Multivitamins in the prevention of cardiovascular disease in men: The Physicians’ Health Study II randomized controlled trial. JAMA.

[19] Wang C., Li Y., Zhu K., Dong Y.M., Sun C.H. (2009). Effects of supplementation with multivitamin and mineral on blood pressure and C-reactive protein in obese Chinese women with increased cardiovascular disease risk. Asia Pac. J. Clin. Nutr..

[20] Age-Related Eye Disease Study 2 Research Group (2013). Lutein + zeaxanthin and omega-3 fatty acids for age-related macular degeneration: The Age-Related Eye Disease Study 2 (AREDS2) randomized clinical trial. JAMA.

[21] Label Claims for Conventional Foods and Dietary Supplements. https://www.fda.gov/Food/IngredientsPackagingLabeling/LabelingNutrition/ucm111447.htm.

[22] Bailey R.L., Gahche J.J., Miller P.E., Thomas P.R., Dwyer J.T. (2013). Why US adults use dietary supplements. JAMA Intern. Med..

[23] Bailey R.L., Akabas S.R., Paxson E.E., Thuppal S.V., Saklani S., Tucker K.L. (2017). Total usual intake of shortfall nutrients varies with poverty among US adults. J. Nutr. Educ. Behav..

[24] Fulgoni V.L., Buckley R.B. (2015). The contribution of fortified ready-to-eat cereal to vitamin and mineral intake in the U.S. population, NHANES 2007–2010. Nutrients.

[25] Schwartz B. (2014). New criteria for supplementation of selected micronutrients in the era of nutrigenetics and nutrigenomics. Int. J. Food Sci. Nutr..

[26] Ferguson L.R., De Caterina R., Gorman U., Allayee H., Kohlmeier M., Prasad C., Choi M.S., Curi R., de Luis D.A., Gil A. (2016). Guide and position of the International Society of Nutrigenetics/Nutrigenomics on personalised nutrition: Part 1—Fields of precision nutrition. J. Nutrigenet. Nutrigenom..

[27] Fenech M., El-Sohemy A., Cahill L., Ferguson L.R., French T.A., Tai E.S., Milner J., Koh W.P., Xie L., Zucker M. (2011). Nutrigenetics and nutrigenomics: Viewpoints on the current status and applications in nutrition research and practice. J. Nutrigenet. Nutrigenom..

[28] Gorman U., Mathers J.C., Grimaldi K.A., Ahlgren J., Nordstrom K. (2013). Do we know enough? A scientific and ethical analysis of the basis for genetic-based personalized nutrition. Genes Nutr..

[29] Nielsen D.E., El-Sohemy A. (2014). Disclosure of genetic information and change in dietary intake: A randomized controlled trial. PLoS ONE.

[30] Price N.D., Magis A.T., Earls J.C., Glusman G., Levy R., Lausted C., McDonald D.T., Kusebauch U., Moss C.L., Zhou Y. (2017). A wellness study of 108 individuals using personal, dense, dynamic data clouds. Nat. Biotechnol..

[31] Goossens J. (2014). Exploring future opportunities and barriers for business model concepts in personalized nutrition. Arch. Public Health.

[32] Grarup N., Sulem P., Sandholt C.H., Thorleifsson G., Ahluwalia T.S., Steinthorsdottir V., Bjarnason H., Gudbjartsson D.F., Magnusson O.T., Sparso T. (2013). Genetic architecture of vitamin B12 and folate levels uncovered applying deeply sequenced large datasets. PLoS Genet..

[33] Jiang X., O’Reilly P.F., Aschard H., Hsu Y.H., Richards J.B., Dupuis J., Ingelsson E., Karasik D., Pilz S., Berry D. (2018). Genome-wide association study in 79,366 European-ancestry individuals informs the genetic architecture of 25-hydroxyvitamin D levels. Nat. Commun..

[34] Kim Y.I. (2003). Role of folate in colon cancer development and progression. J. Nutr..

[35] Levine A.J., Figueiredo J.C., Lee W., Conti D.V., Kennedy K., Duggan D.J., Poynter J.N., Campbell P.T., Newcomb P., Martinez M.E. (2010). A candidate gene study of folate-associated one carbon metabolism genes and colorectal cancer risk. Cancer Epidemiol. Biomark. Prev..

[36] Figueiredo J.C., Levine A.J., Lee W.H., Conti D.V., Poynter J.N., Campbell P.T., Duggan D., Lewinger J.P., Martinez M.E., Ulrich C.M. (2010). Genes involved with folate uptake and distribution and their association with colorectal cancer risk. Cancer Causes Control.

[37] Cheng T.Y., Makar K.W., Neuhouser M.L., Miller J.W., Song X., Brown E.C., Beresford S.A., Zheng Y., Poole E.M., Galbraith R.L. (2015). Folate-mediated one-carbon metabolism genes and interactions with nutritional factors on colorectal cancer risk: Women’s Health Initiative Observational Study. Cancer.

[38] Berry R.J., Li Z., Erickson J.D., Li S., Moore C.A., Wang H., Mulinare J., Zhao P., Wong L.Y., Gindler J. (1999). Prevention of neural-tube defects with folic acid in China. China-U.S. Collaborative Project for Neural Tube Defect Prevention. N. Engl. J. Med..

[39] MRC Vitamin Study Research Group (1991). Prevention of neural tube defects: Results of the Medical Research Council Vitamin Study. Lancet.

[40] Branum A.M., Bailey R., Singer B.J. (2013). Dietary supplement use and folate status during pregnancy in the United States. J. Nutr..

[41] Choi S.W., Mason J.B. (2002). Folate status: Effects on pathways of colorectal carcinogenesis. J. Nutr..

[42] Seshadri S., Beiser A., Selhub J., Jacques P.F., Rosenberg I.H., D’Agostino R.B., Wilson P.W., Wolf P.A. (2002). Plasma homocysteine as a risk factor for dementia and Alzheimer’s disease. N. Engl. J. Med..

[43] Kim Y.I. (1999). Folate and carcinogenesis: Evidence, mechanisms, and implications. J. Nutr. Biochem..

[44] Kim Y.I. (2000). Methylenetetrahydrofolate reductase polymorphisms, folate, and cancer risk: A paradigm of gene-nutrient interactions in carcinogenesis. Nutr. Rev..

[45] Homocysteine Studies Collaboration (2002). Homocysteine and risk of ischemic heart disease and stroke: A meta-analysis. JAMA.

[46] Boushey C.J., Beresford S.A., Omenn G.S., Motulsky A.G. (1995). A quantitative assessment of plasma homocysteine as a risk factor for vascular disease. Probable benefits of increasing folic acid intakes. JAMA.

[47] Ray J.G. (1998). Meta-analysis of hyperhomocysteinemia as a risk factor for venous thromboembolic disease. Arch. Intern. Med..

[48] Kim J., Cho Y.A., Kim D.H., Lee B.H., Hwang D.Y., Jeong J., Lee H.J., Matsuo K., Tajima K., Ahn Y.O. (2012). Dietary intake of folate and alcohol, MTHFR C677T polymorphism, and colorectal cancer risk in Korea. Am. J. Clin. Nutr..

[49] O’Reilly S.L., McGlynn A.P., McNulty H., Reynolds J., Wasson G.R., Molloy A.M., Strain J.J., Weir D.G., Ward M., McKerr G. (2016). Folic acid supplementation in postpolypectomy patients in a randomized controlled trial increases tissue folate concentrations and reduces aberrant DNA biomarkers in colonic tissues adjacent to the former polyp site. J. Nutr..

[50] Svensson T., Yamaji T., Budhathoki S., Hidaka A., Iwasaki M., Sawada N., Inoue M., Sasazuki S., Shimazu T., Tsugane S. (2016). Alcohol consumption, genetic variants in the alcohol- and folate metabolic pathways and colorectal cancer risk: The JPHC Study. Sci. Rep..

[51] Chen J., Giovannucci E., Kelsey K., Rimm E.B., Stampfer M.J., Colditz G.A., Spiegelman D., Willett W.C., Hunter D.J. (1996). A methylenetetrahydrofolate reductase polymorphism and the risk of colorectal cancer. Cancer Res..

[52] Ma J., Stampfer M.J., Giovannucci E., Artigas C., Hunter D.J., Fuchs C., Willett W.C., Selhub J., Hennekens C.H., Rozen R. (1997). Methylenetetrahydrofolate reductase polymorphism, dietary interactions, and risk of colorectal cancer. Cancer Res..

[53] Slattery M.L., Potter J.D., Samowitz W., Schaffer D., Leppert M. (1999). Methylenetetrahydrofolate reductase, diet, and risk of colon cancer. Cancer Epidemiol. Biomark. Prev..

[54] Ulrich C.M., Kampman E., Bigler J., Schwartz S.M., Chen C., Bostick R., Fosdick L., Beresford S.A., Yasui Y., Potter J.D. (1999). Colorectal adenomas and the C677T MTHFR polymorphism: Evidence for gene-environment interaction?. Cancer Epidemiol. Biomark. Prev..

[55] Maruti S.S., Ulrich C.M., Jupe E.R., White E. (2009). MTHFR C677T and postmenopausal breast cancer risk by intakes of one-carbon metabolism nutrients: A nested case-control study. Breast Cancer Res..

[56] Cole B.F., Baron J.A., Sandler R.S., Haile R.W., Ahnen D.J., Bresalier R.S., McKeown-Eyssen G., Summers R.W., Rothstein R.I., Burke C.A. (2007). Folic acid for the prevention of colorectal adenomas: A randomized clinical trial. JAMA.

[57] Figueiredo J.C., Grau M.V., Haile R.W., Sandler R.S., Summers R.W., Bresalier R.S., Burke C.A., McKeown-Eyssen G.E., Baron J.A. (2009). Folic acid and risk of prostate cancer: Results from a randomized clinical trial. J. Natl. Cancer Inst..

[58] Bae S., Ulrich C.M., Bailey L.B., Malysheva O., Brown E.C., Maneval D.R., Neuhouser M.L., Cheng T.Y., Miller J.W., Zheng Y. (2014). Impact of folic acid fortification on global DNA methylation and one-carbon biomarkers in the Women’s Health Initiative Observational Study cohort. Epigenetics.

[59] Miller J.W., Ulrich C.M. (2013). Folic acid and cancer—Where are we today?. Lancet.

[60] Neuhouser M.L., Nijhout H.F., Gregory J.F., Reed M.C., James S.J., Liu A., Shane B., Ulrich C.M. (2011). Mathematical modeling predicts the effect of folate deficiency and excess on cancer-related biomarkers. Cancer Epidemiol. Biomark. Prev..

[61] Ulrich C.M., Toriola A.T., Koepl L.M., Sandifer T., Poole E.M., Duggan C., McTiernan A., Issa J.P. (2012). Metabolic, hormonal and immunological associations with global DNA methylation among postmenopausal women. Epigenetics.

[62] Troen A.M., Mitchell B., Sorensen B., Wener M.H., Johnston A., Wood B., Selhub J., McTiernan A., Yasui Y., Oral E. (2006). Unmetabolized folic acid in plasma is associated with reduced natural killer cell cytotoxicity among postmenopausal women. J. Nutr..

[63] Sawaengsri H., Wang J., Reginaldo C., Steluti J., Wu D., Meydani S.N., Selhub J., Paul L. (2016). High folic acid intake reduces natural killer cell cytotoxicity in aged mice. J. Nutr. Biochem..

[64] Abbenhardt C., Miller J.W., Song X., Brown E.C., Cheng T.Y., Wener M.H., Zheng Y., Toriola A.T., Neuhouser M.L., Beresford S.A. (2014). Biomarkers of one-carbon metabolism are associated with biomarkers of inflammation in women. J. Nutr..

[65] Matsha T.E., Pheiffer C., Humphries S.E., Gamieldien J., Erasmus R.T., Kengne A.P. (2016). Genome-wide DNA methylation in mixed ancestry individuals with diabetes and prediabetes from South Africa. Int. J. Endocrinol..

[66] Joubert B.R., den Dekker H.T., Felix J.F., Bohlin J., Ligthart S., Beckett E., Tiemeier H., van Meurs J.B., Uitterlinden A.G., Hofman A. (2016). Maternal plasma folate impacts differential DNA methylation in an epigenome-wide meta-analysis of newborns. Nat. Commun..

[67] Troesch B., Weber P., Mohajeri M.H. (2016). Potential links between impaired one-carbon metabolism due to polymorphisms, inadequate B-vitamin status, and the development of Alzheimer’s disease. Nutrients.

[68] Philip D., Buch A., Moorthy D., Scott T.M., Parnell L.D., Lai C.Q., Ordovas J.M., Selhub J., Rosenberg I.H., Tucker K.L. (2015). Dihydrofolate reductase 19-bp deletion polymorphism modifies the association of folate status with memory in a cross-sectional multi-ethnic study of adults. Am. J. Clin. Nutr..

[69] Lai C.Q., Parnell L.D., Troen A.M., Shen J., Caouette H., Warodomwichit D., Lee Y.C., Crott J.W., Qiu W.Q., Rosenberg I.H. (2010). MAT1A variants are associated with hypertension, stroke, and markers of DNA damage and are modulated by plasma vitamin B-6 and folate. Am. J. Clin. Nutr..

[70] Davis C.D., Milner J.A. (2011). Nutrigenomics, vitamin D and cancer prevention. J. Nutrigenet. Nutrigenom..

[71] Kriebitzsch C., Verlinden L., Eelen G., Tan B.K., Van Camp M., Bouillon R., Verstuyf A. (2009). The impact of 1,25(OH)2D3 and its structural analogs on gene expression in cancer cells—A microarray approach. Anticancer Res..

[72] Patrick R.P., Ames B.N. (2015). Vitamin D and the omega-3 fatty acids control serotonin synthesis and action, part 2: Relevance for ADHD, bipolar disorder, schizophrenia, and impulsive behavior. FASEB J..

[73] Patrick R.P., Ames B.N. (2014). Vitamin D hormone regulates serotonin synthesis. Part 1: Relevance for autism. FASEB J..

[74] Rossom R.C., Espeland M.A., Manson J.E., Dysken M.W., Johnson K.C., Lane D.S., LeBlanc E.S., Lederle F.A., Masaki K.H., Margolis K.L. (2012). Calcium and vitamin D supplementation and cognitive impairment in the Women’s Health Initiative. J. Am. Geriatr. Soc..

[75] Kjaergaard M., Waterloo K., Wang C.E., Almas B., Figenschau Y., Hutchinson M.S., Svartberg J., Jorde R. (2012). Effect of vitamin D supplement on depression scores in people with low levels of serum 25-hydroxyvitamin D: Nested case-control study and randomised clinical trial. Br. J. Psychiatry.

[76] Zanon-Moreno V., Asensio-Marquez E.M., Ciancotti-Oliver L., Garcia-Medina J.J., Sanz P., Ortega-Azorin C., Pinazo-Duran M.D., Ordovas J.M., Corella D. (2013). Effects of polymorphisms in vitamin E-, vitamin C-, and glutathione peroxidase-related genes on serum biomarkers and associations with glaucoma. Mol. Vis..

[77] Belisle S.E., Hamer D.H., Leka L.S., Dallal G.E., Delgado-Lista J., Fine B.C., Jacques P.F., Ordovas J.M., Meydani S.N. (2010). IL-2 and IL-10 gene polymorphisms are associated with respiratory tract infection and may modulate the effect of vitamin E on lower respiratory tract infections in elderly nursing home residents. Am. J. Clin. Nutr..

[78] Belisle S.E., Leka L.S., Delgado-Lista J., Jacques P.F., Ordovas J.M., Meydani S.N. (2009). Polymorphisms at cytokine genes may determine the effect of vitamin E on cytokine production in the elderly. J. Nutr..

[79] Gibson R.S. (2007). The role of diet- and host-related factors in nutrient bioavailability and thus in nutrient-based dietary requirement estimates. Food Nutr. Bull..

[80] Pfeiffer C.M., Sternberg M.R., Schleicher R.L., Rybak M.E. (2013). Dietary supplement use and smoking are important correlates of biomarkers of water-soluble vitamin status after adjusting for sociodemographic and lifestyle variables in a representative sample of U.S. adults. J. Nutr..

[81] Lam J.R., Schneider J.L., Quesenberry C.P., Corley D.A. (2017). Proton pump inhibitor and histamine-2 receptor antagonist use and iron deficiency. Gastroenterology.

[82] Aroda V.R., Edelstein S.L., Goldberg R.B., Knowler W.C., Marcovina S.M., Orchard T.J., Bray G.A., Schade D.S., Temprosa M.G., White N.H. (2016). Long-term metformin use and vitamin B12 deficiency in the diabetes prevention program outcomes study. J. Clin. Endocrinol. Metab..

[83] Lam J.R., Schneider J.L., Zhao W., Corley D.A. (2013). Proton pump inhibitor and histamine 2 receptor antagonist use and vitamin B12 deficiency. JAMA.

[84] Namaste S.M., Aaron G.J., Varadhan R., Peerson J.M., Suchdev P.S. (2017). Methodologic approach for the Biomarkers Reflecting Inflammation and Nutritional Determinants of Anemia (BRINDA) project. Am. J. Clin. Nutr..

[85] Ogden C.L., Carroll M.D., Fryar C.D., Flegal K.M. (2015). Prevalence of obesity among adults and youth: United States, 2011–2014. NCHS Data Brief.

[86] Peterson L.A., Cheskin L.J., Furtado M., Papas K., Schweitzer M.A., Magnuson T.H., Steele K.E. (2016). Malnutrition in bariatric surgery candidates: Multiple micronutrient deficiencies prior to surgery. Obes. Surg..

[87] Pereira-Santos M., Costa P.R., Assis A.M., Santos C.A., Santos D.B. (2015). Obesity and vitamin D deficiency: A systematic review and meta-analysis. Obes. Rev..

[88] Maffoni S., De Giuseppe R., Stanford F.C., Cena H. (2017). Folate status in women of childbearing age with obesity: A review. Nutr. Res. Rev..

[89] Watkins M.L., Erickson J.D., Thun M.J., Mulinare J., Heath C.W. (2000). Multivitamin use and mortality in a large prospective study. Am. J. Epidemiol..

[90] Holmquist C., Larsson S., Wolk A., de Faire U. (2003). Multivitamin supplements are inversely associated with risk of myocardial infarction in men and women—Stockholm Heart Epidemiology Program (SHEEP). J. Nutr..

[91] Rautiainen S., Akesson A., Levitan E.B., Morgenstern R., Mittleman M.A., Wolk A. (2010). Multivitamin use and the risk of myocardial infarction: A population-based cohort of Swedish women. Am. J. Clin. Nutr..

[92] Neuhouser M.L., Wassertheil-Smoller S., Thomson C., Aragaki A., Anderson G.L., Manson J.E., Patterson R.E., Rohan T.E., Van H.L., Shikany J.M. (2009). Multivitamin use and risk of cancer and cardiovascular disease in the Women’s Health Initiative cohorts. Arch. Intern. Med..

[93] Bailey R.L., Fakhouri T.H., Park Y., Dwyer J.T., Thomas P.R., Gahche J.J., Miller P.E., Dodd K.W., Sempos C.T., Murray D.M. (2015). Multivitamin-mineral use is associated with reduced risk of cardiovascular disease mortality among women in the United States. J. Nutr..

[94] Park S.Y., Murphy S.P., Wilkens L.R., Henderson B.E., Kolonel L.N. (2011). Multivitamin use and the risk of mortality and cancer incidence: The Multiethnic Cohort Study. Am. J. Epidemiol..

[95] Jacobs E.J., Connell C.J., Patel A.V., Chao A., Rodriguez C., Seymour J., McCullough M.L., Calle E.E., Thun M.J. (2001). Multivitamin use and colon cancer mortality in the Cancer Prevention Study II cohort (United States). Cancer Causes Control.

[96] Rautiainen S., Rist P.M., Glynn R.J., Buring J.E., Gaziano J.M., Sesso H.D. (2016). Multivitamin use and the risk of cardiovascular disease in men. J. Nutr..

[97] Blot W.J., Li J.Y., Taylor P.R., Guo W., Dawsey S., Wang G.Q., Yang C.S., Zheng S.F., Gail M., Li G.Y. (1993). Nutrition intervention trials in Linxian, China: Supplementation with specific vitamin/mineral combinations, cancer incidence, and disease-specific mortality in the general population. J. Natl. Cancer Inst..

[98] Li J.Y., Taylor P.R., Li B., Dawsey S., Wang G.Q., Ershow A.G., Guo W., Liu S.F., Yang C.S., Shen Q. (1993). Nutrition intervention trials in Linxian, China: Multiple vitamin/mineral supplementation, cancer incidence, and disease-specific mortality among adults with esophageal dysplasia. J. Natl. Cancer Inst..

[99] Christen W.G., Glynn R.J., Manson J.E., MacFadyen J., Bubes V., Schvartz M., Buring J.E., Sesso H.D., Gaziano J.M. (2014). Effects of multivitamin supplement on cataract and age-related macular degeneration in a randomized trial of male physicians. Ophthalmology.

[100] Gaziano J.M., Sesso H.D., Christen W.G., Bubes V., Smith J.P., MacFadyen J., Schvartz M., Manson J.E., Glynn R.J., Buring J.E. (2012). Multivitamins in the prevention of cancer in men. The Physicians’ Health Study II randomized controlled trial. JAMA.

[101] Hercberg S., Galan P., Preziosi P., Bertrais S., Mennen L., Malvy D., Roussel A.M., Favier A., Briancon S. (2004). The SU.VI.MAX Study: A randomized, placebo-controlled trial of the health effects of antioxidant vitamins and minerals. Arch. Intern. Med..

[102] Lamas G.A., Boineau R., Goertz C., Mark D.B., Rosenberg Y., Stylianou M., Rozema T., Nahin R.L., Lindblad L., Lewis E.F. (2013). Oral high-dose multivitamins and minerals after myocardial infarction: A randomized trial. Ann. Intern. Med..

[103] AREDS (2001). A randomized, placebo-controlled, clinical trial of high-dose supplementation with vitamins C and E, beta carotene, and zinc for age-related macular degeneration and vision loss: AREDS report no. 8. Arch. Ophthalmol..

[104] AREDS (2001). A randomized, placebo-controlled, clinical trial of high-dose supplementation with vitamins C and E and beta carotene for age-related cataract and vision loss: AREDS report no. 9. Arch. Ophthalmol..

[105] Chew E.Y., SanGiovanni J.P., Ferris F.L., Wong W.T., Agron E., Clemons T.E., Sperduto R., Danis R., Chandra S.R., Blodi B.A. (2013). Lutein/zeaxanthin for the treatment of age-related cataract: AREDS2 randomized trial report no. 4. JAMA Ophthalmol..

[106] Assmann K.E., Andreeva V.A., Jeandel C., Hercberg S., Galan P., Kesse-Guyot E. (2015). Healthy aging 5 years after a period of daily supplementation with antioxidant nutrients: A post hoc analysis of the French randomized trial SU.VI.MAX. Am. J. Epidemiol..

[107] Rautiainen S., Gaziano J.M., Christen W.G., Bubes V., Kotler G., Glynn R.J., Manson J.E., Buring J.E., Sesso H.D. (2017). Effect of baseline nutritional status on long-term multivitamin use and cardiovascular disease risk: A secondary analysis of the Physicians’ Health Study II randomized clinical trial. JAMA Cardiol..

[108] Biesalski H.K., Tinz J. (2017). Multivitamin/mineral supplements: Rationale and safety—A systematic review. Nutrition.

[109] Macpherson H., Pipingas A., Pase M.P. (2013). Multivitamin-multimineral supplementation and mortality: A meta-analysis of randomized controlled trials. Am. J. Clin. Nutr..

[110] Fortmann S.P., Burda B.U., Senger C.A., Lin J.S., Whitlock E.P. (2013). Vitamin and mineral supplements in the primary prevention of cardiovascular disease and cancer: An updated systematic evidence review for the U.S. Preventive Services Task Force. Ann. Intern. Med..

[111] COcoa Supplement and Multivitamin Outcomes Study (COSMOS) NCT02422745. NCT02422745.

